# Study on D6AC Steel PCBN Hard Turning and Optimization

**DOI:** 10.3390/ma19091850

**Published:** 2026-04-30

**Authors:** Yihan Liu, Shutao Huang, Ruyu Li, Zhonghan Cui, Yupu Zhang, Chengwei Liu

**Affiliations:** 1 Laboratory Management Center, Shenyang Ligong University, Shenyang 110159, China; 2 School of Mechanical Engineering, Shenyang Ligong University, Shenyang 110159, China; 3 Innovation and Entrepreneurship College, Shenyang Ligong University, Shenyang 110159, China

**Keywords:** D6AC, PCBN, hard turning, processes, ANOVA, GRA

## Abstract

This investigation uses polycrystalline cubic boron nitride (PCBN) tools for precision turning of D6AC (45CrNiMoVA) hardened steel, thereby enabling the manufacturing of components that meet the requirements of intelligent manufacturing lines. A Taguchi’s L16 (4^3^) orthogonal design was employed to systematically investigate the effects of cutting speed, depth of cut, and feed rate on cutting force, cutting temperature, surface roughness, and tool wear. Analysis of variance (ANOVA) was then conducted to quantify the contribution of each cutting parameter, and high-accuracy predictive models (R^2^ > 0.86) were established for the key response variables, namely cutting force components (*F_x_*, *F_y_*, *F_z_*), cutting temperature (*T*), and flank wear width (*VB_max_*). The results show that excellent surface quality can be achieved within the investigated range, namely at cutting speeds of 100–250 m·min^−1^, depths of cut of 0.05–0.2 mm, and feed rates of 0.05–0.125 mm·rev^−1^, with surface roughness (*Ra*) below 0.8 μm and mostly around 0.4 µm. At a feed rate of 0.05 mm·rev^−1^, the measured Ra was greater than the theoretical value (Ra*), whereas at a feed rate of 0.075 mm·rev^−1^, *Ra* was lower than Ra*, with the difference increasing as feed rate increased. The ANOVA results showed that cutting forces were dominated by depth of cut, cutting temperatures by feed rate, and tool wear by depth of cut. The optimal process strategy was derived as follows: first, prioritize a lower feed rate; second, select an appropriate depth of cut based on tool failure or deformation control objectives; and third, choose a suitable cutting speed according to tool-life requirements or machining efficiency. This study provides process guidance and predictive tools for PCBN finishing of D6AC steel, thus promoting green, precise, and efficient machining of high-strength, high-hardness, and low-thermal-conductivity materials.

## 1. Introduction

The D6AC (45CrNiMoVA) steel is widely used in engineering practice, especially in aerospace and related industries, because it has high strength, outstanding fracture toughness, and stress corrosion resistance [1,2]. In production, components typically undergo precision machining after quenching. However, quenching increases strength and hardness while reducing ductility and thermal conductivity, leading to higher cutting forces, rapid tool wear, and heat accumulation. These issues complicate machining, limit efficiency and quality, and constitute critical bottlenecks in advanced manufacturing sectors such as aerospace. Given the ongoing transition toward green and intelligent manufacturing, efficient and environmentally friendly precision-machining technologies for this material must be compatible with the requirements of intelligent production lines.

Grinding is the conventional finishing method for hardened steel, but it is inefficient, generates high temperatures, produces significant residual stresses, and causes deformation [3]. By contrast, replacing grinding with turning enables the finishing of parts with complex contours, facilitates integration with intelligent production lines, improves machining efficiency, and reduces energy consumption [4,5]. Research into the mechanisms and processes of hard turning for difficult-to-machine materials is of great significance, advancing the industrialization of the “turning instead of grinding” concept.

Polycrystalline cubic boron nitride (PCBN) tools are well suited for machining hardened steel because of their outstanding wear resistance, high hardness, superior thermal conductivity, and chemical inertness [6,7,8,9]. Wang et al. [10] developed a binderless nanopolycrystalline PCBN end mill, and grey relational analysis (GRA) indicated that cutting temperature (*T*) significantly influenced tool wear. Osička et al. [11] achieved Ra values of 0.3–0.4 μm when hard turning 100Cr6 with PCBN, demonstrating that hard turning can replace grinding to obtain superior surfaces. Xu et al. [12] found that PCBN tools outperformed PCD tools under high-load hard machining conditions when milling 3Y-TZP ceramics.

The results of hard turning stem from the combined effects of cutting parameters, and understanding their influence patterns is fundamental to optimizing the process. Numerous studies indicate that feed rate (*f*) and depth of cut (*a_p_*) exert decisive effects on cutting forces, while cutting speed (*v*) has a negligible impact. Kumar et al. [13] found that feed rate has the primary effect on the main cutting force in the turning of AISI D2 hardened steel, while cutting speed most strongly influences surface roughness (*Ra*). Tzotzis et al. [14] used finite element simulation to analyze ceramic tool turning of AISI-52100 hardened steel, finding depth of cut and feed rate exerted the greatest influence on cutting forces; Prasad et al. [15] observed that increasing cutting speed exacerbated front-face crater wear and affected *Ra* during hard turning of Incoloy 800 steel. Additionally, Bartarya et al. [16] revealed the relationship between tool wear and surface quality, demonstrating that feed rate and depth of cut affect machining outcomes by altering cutting loads. Moreover, cooling and lubrication strategies are crucial for controlling cutting forces and tool wear [17,18].

Performance prediction and process parameter optimization are central to enhancing the level of hard turning process systems. For instance, predicting tool wear enables the monitoring of tool status on production lines [19]. Abbas et al. [20] compared various algorithms for hard turning AISI 1045 steel to achieve maximum machining efficiency and minimum surface roughness. Among these, grey relational analysis (GRA) combined with the Taguchi method is widely applied in multi-objective optimization [21,22]. This methodology has been used by Zhujani et al. [23] to study hard turning on Inconel 718 steel, where the importance of parameters was assessed by ANOVA to enable the extraction of parameter combinations that are efficient and of high quality. Ponugoti et al. [24] employed a GRA-PCA hybrid method for multi-objective optimization of cutting forces, cutting temperature, and surface roughness to determine the optimal process scheme. Additionally, some studies adopted a holistic perspective on process influence mechanisms. For instance, the effects of cutting speed on main cutting force and surface roughness of the workpiece surface in KhVG steel’s hard turning were discussed by Stakhniv et al. [25].

However, existing studies still exhibit several limitations. First, most investigations focus on conventional hardened steels, whereas D6AC has received relatively little attention. Its unique properties after quenching necessitate dedicated machining strategies. Second, current research predominantly addresses single-objective trends rather than simultaneously resolving issues related to cutting force, temperature, tool wear, and material removal rate. Third, the lack of predictive models for the performance of PCBN tools in the precision turning of D6AC restricts process planning and decision-making in industrial applications.

To address these issues, this study investigates the cutting mechanisms and process optimization of precision hard turning of quenched D6AC steel using PCBN tools. Experimental cost was minimized through a Taguchi orthogonal design. The effects of cutting parameters on cutting force, cutting temperature, surface roughness, and tool wear were quantified using ANOVA. Multi-objective optimization based on response surface methodology (RSM) and GRA was then performed to achieve simultaneous performance improvements while maintaining surface quality. Optimal parameter combinations and quantitative correlation models were obtained. Accordingly, this study establishes an integrated framework for mechanism analysis, performance prediction, and process optimization in the precision hard turning of D6AC steel with PCBN tools. It addresses the lack of cutting data and mechanistic understanding and provides production-oriented parameter guidance. These findings improve machining efficiency and product quality while controlling production costs, thereby promoting intelligent and green manufacturing of precision structural components in high-end industries.

## 2. Materials and Methods

**Figure 1:**
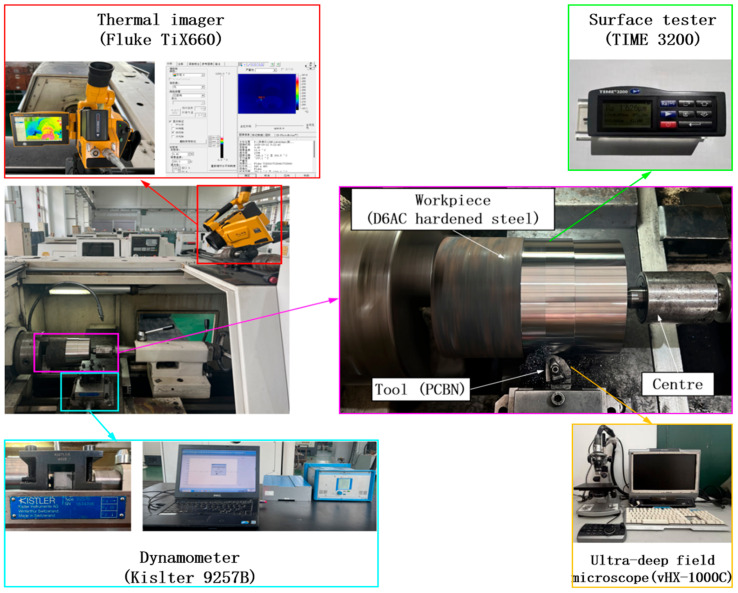
Experimental setup.

As illustrated in Figure 1, the turning experiments were carried out on a CAK3665nzi CNC lathe (Shenyang Machine Tool Co., Ltd., Shenyang, China). The main technical specifications of the machine are as follows: maximum spindle speed, 3000 r/min; spindle power, 7.5 kW; maximum turning diameter, 180 mm; maximum turning length, 650 mm; positioning accuracy/repeatability, 0.04/0.016 mm; and with a lathe tool shank cross-section of 20 mm × 20 mm. These specifications ensured stable execution of the hard-turning experiments under the selected finishing conditions. A hardened D6AC (45CrNiMoVA) steel cylindrical workpiece (φ145 mm) was machined using PCBN tools. The chemical composition is based on the material certificate provided by the steel supplier, as shown in Table 1. The material properties of the hardened D6AC steel based on measurements are summarized in Table 2. The turning experiments were carried out using uncoated solid polycrystalline cubic boron nitride (PCBN) inserts (ISCAR Ltd., Tefen, Israel). The insert ordering code was WNGA 080404T-MC (80° rhombic shape, negative rake, 12.7 mm inscribed circle, 4.76 mm thickness, 0.4 mm nose radius) with grade IB55 (high CBN content, H10–H25/K05–K15 ISO application range). Based on the manufacturer’s recommendations for hard turning of hardened steel, the cutting parameters were selected as follows: cutting speed *v* = 120–200 m/min, feed rate *f* = 0.05–0.2 mm/r, and depth of cut *a_p_* = 0.05–0.5 mm. The experimental parameter window used in this study was selected within or with reference to these recommended ranges, and its detailed geometric parameters are provided in Table 3.

**Figure 2:**
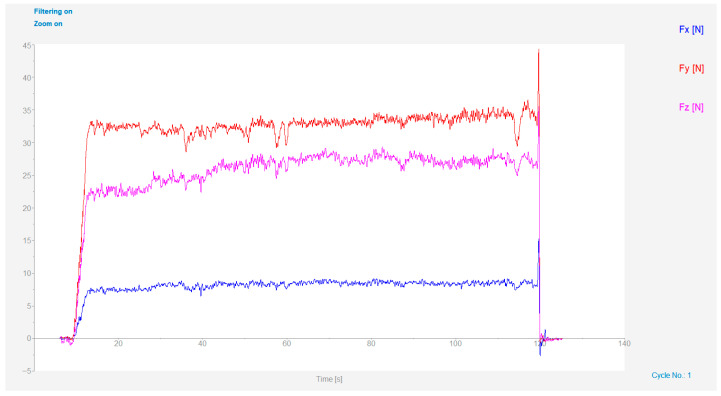
Waveforms of cutting force signals (*v* = 150 m**·**min^−1^, *a_p_* = 0.05 mm, *f* = 0.075 mm**·**rev^−1^).

The cutting force measurement system consisted of a 9257B plate dynamometer (Kistler, Winterthur, Switzerland), a 5070A charge amplifier, a 5697A data acquisition unit, and a computer, with a sampling frequency set to 250 Hz. The cutting force signal waveforms were processed by removing drift and filtering, and the data were acquired and processed using DynoWare software (Version 2825A, Kistler Group, Winterthur, Switzerland). The average values were used to calibrate the main cutting force (*F_z_*), radial force (*F_y_*), and axial force (*F_x_*), as shown in Figure 2. The resultant cutting force was calculated as Equation (1).

(1) $$F_{\sum }=\sqrt{{F_{x}}^{2}+{F_{y}}^{2}+{F_{z}}^{2}}$$

where *F_z_* (N) is the main force, *F_y_* (N) is the radial force, and *F_x_* (N) is the axial force.

**Figure 3:**
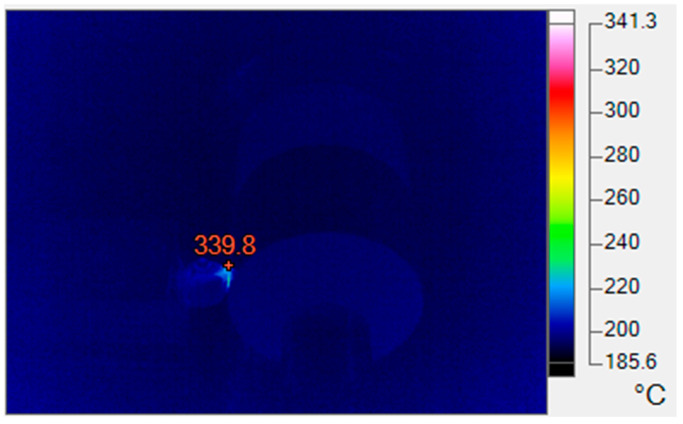
Thermal imaging of the cutting zone (*v* = 150 m**·**min^−1^, *a_p_* = 0.1 mm, f = 0.05 mm**·**rev^−1^).

The temperature field was captured using the Fluke TiX660 thermal imager (Fluke Corp., Everett, WA, USA) with a temperature range of 250 to 1200 °C. The emissivity of D6AC hardened steel was calibrated experimentally at 0.4. Since cutting is continuous and the thin chips flow along the rake face, the actual cutting temperature is difficult to measure. Therefore, the highest temperature observed near the tool tip within the visible field of the thermal imager was selected as the representative cutting temperature. During each experiment, the cutting temperature was continuously monitored. In the absence of chip interference within the field of view, the highest temperature in the visible area was recorded as a single measurement. Five such measurements were taken per cutting condition; after removing outliers, the average value was calculated and reported as *T* (°C). This value provides a practical reference for analyzing the actual machining process and for selecting cutting parameters, as illustrated in Figure 3.

**Figure 4:**
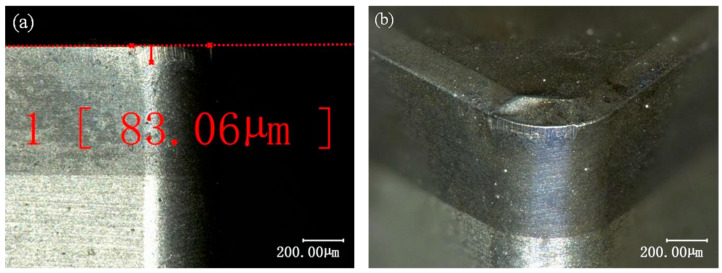
Morphology of tool (*v* = 150 m**·**min^−1^, *a_p_* = 0.1 mm, *f* = 0.05 mm**·**rev^−1^). (**a**) The flank face (**b**) The cutting edge.

The flank wear of the cutting tool was observed using a digital depth-of-field microscope (KEYENCE VHX-1000C, Keyence Corporation, Osaka, Japan). As shown in Figure 4a, at the position where the wear was maximum, five repeated measurements were performed. After removing outliers, the average value was taken as the maximum wear width of the flank face, *VB_max_* (μm).

The surface roughness *Ra* (μm) was evaluated using a TIME 3200 profilometer (Beijing TIME High Technology Ltd., Beijing, China) with a sampling length of 0.8 mm, and seven points were randomly selected on the machined surface. Outliers were identified and removed, and the remaining values were averaged to obtain the final reported measurement.

The material removal rate (MRR) was used to characterize machining efficiency, calculated as Equation (2).

(2) $$MRR=1000vfa_{p}$$

where *v* (m·min^−1^) is cutting speed, *a_p_* (mm) is depth of cut, *f* (mm·rev^−1^) is feed rate.

The experiments were performed by means of the Taguchi L_16_(4^3^) design of experiments. Table 4 presents the cutting parameter factors and level settings obtained through orthogonal experiment analysis. Under dry cutting conditions, the axial cutting length for each test was set to 45 mm.

## 3. Results and Discussion

The design and measurement results of the orthogonal experiments are presented in Table 4. The cutting-parameter levels were selected based on the tool manufacturer’s recommended range, relevant literature on PCBN hard turning of hardened steels, and preliminary trial cutting. The predictive models were fitted using the L16 orthogonal experimental data and evaluated by comparing the predicted and measured response values; model adequacy was assessed using R^2^ and ANOVA. No independent external validation dataset was used.

The data reveal that 12 out of 16 experiments exhibited *F_y_* > *F_z_* > *F_x_*, when *a_p_* = 0.05–0.15 mm; the remaining at *a_p_* = 0.2 mm showed *F_z_* > *F_y_* > *F_x_*. The variation in cutting force component magnitudes results from the combined effects of the actual conditions along the arc-shaped cutting edge at varying depth of cut, the degree of metal deformation, and the patterns of force decomposition.

**Figure 5:**
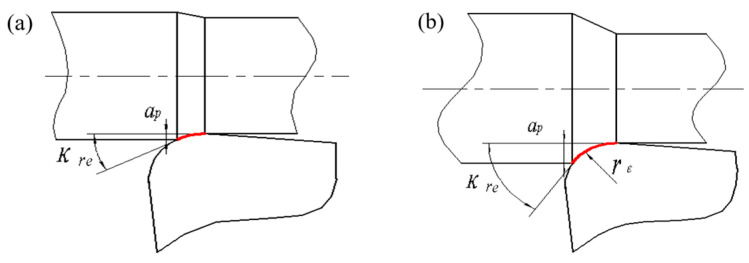
The engaged cutting edge under varying *a_p_* (**a**) *a_p_* = 0.05 mm (**b**) *a_p_* = 0.2 mm.

As illustrated in Figure 5, the engaged cutting-edge arc length increases with depth of cut, causing a variation in actual cutting-edge angle *κ_re_*. Increasing depth of cut expands width of cut *a_w_* (the engaged width of the uncut layer along the major cutting edge, and it can be expressed as *a_w_* = *a_p_*/sin*κ_re_*), while a rise in actual cutting-edge angle *κ_re_* enhances the undeformed chip thickness *a_c_*. The interplay between *a_w_* and *a_c_* controls the development of the resolved cutting forces. Concurrently, the temperature field in the shear zones affects the frictional coefficients, thereby influencing the main cutting force *F_z_*. Cutting forces arise from the resistance of the workpiece to elastic–plastic deformation, together with friction at the tool–workpiece and tool–chip interfaces. The magnitudes of the three orthogonal components are determined by the actual cutting-edge angle *κ_re_* and the degree of cutting deformation.

At *a_p_* = 0.05–0.15 mm, despite an increase in actual cutting-edge angle *κ_re_*, the small depth of cut keeps the additional engaged arc segment at *κ_re_* < 45°. Under this condition, *a_w_* > *a_c_*, and the tool–workpiece contact area expands primarily radially. This radially concentrated plastic deformation causes the radial deformation resistance to increase more significantly with depth of cut. At *a_p_* = 0.2 mm, the actual cutting-edge angle *κ_re_* exceeds 45°, further extending the engaged arc length, which moderates the increment of the radial force *F_y_*. Meanwhile, the main cutting force *F_z_* continues to rise with the expanding engaged arc length. Furthermore, as analyzed later on regarding cutting temperature, a larger depth of cut *a_p_* will generate more heat, resulting in a high temperature concentration at the cutting position and increasing the friction coefficient. Since the main cutting force *F_z_* must overcome deformation and friction in the cutting speed direction, its increase is more pronounced than that of radial force *F_y_*.

### 3.1. Development of Predictive Models

Using the Matlab software (Version R2021b, The MathWorks Inc., Natick, MA, USA) and employing a multivariate nonlinear regression method, predictive models for various cutting performance indicators were established, as shown in Equations (3)–(5). A power-law form was adopted because cutting force, cutting temperature, and tool wear generally exhibit nonlinear multiplicative relationships with cutting speed, depth of cut, and feed rate. This model form is widely used in machining studies, as it can effectively capture parameter coupling effects while retaining a simple structure and clear physical interpretability.

(3) $$\left\{\begin{array}{c} F_{x}=365.85v^{0.12}{a_{p}}^{1.27}f^{0.25}\\ F_{y}=377.79v^{0.18}{a_{p}}^{0.77}f^{0.42}\\ F_{z}=497.08v^{0.17}{a_{p}}^{0.94}f^{0.40} \end{array}\right.$$

(4) $$T=512.20v^{-0.01}{a_{p}}^{0.05}f^{0.10}$$

(5) $${VB}_{max}=58.83v^{0.12}{a_{p}}^{-0.14}f^{0.18}$$

The calculation results of coefficients of determination (R^2^) and mean relative error (MRE) are presented in Table 5. R^2^ for each output exceeds 0.86, MRE for all predictions is below 10%, indicating strong explanatory power of the models. In particular, the prediction for axial force *F_x_* achieves the highest goodness-of-fit (R^2^ ≈ 0.97).

### 3.2. Influence Law of Cutting Parameters

The theoretical surface roughness Ra* (μm) is calculated via Equation (6) [26].

(6) $${Ra}^{*}=\frac{f^{2}}{18\sqrt{3}r_{\varepsilon }}$$

where *r_ε_* (mm) is corner radius.

**Figure 6:**
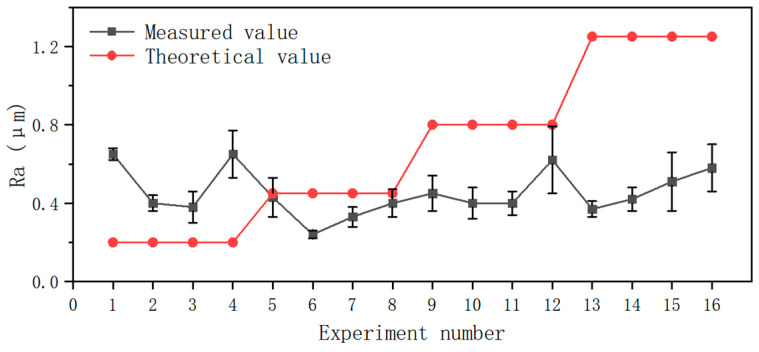
Comparison between theoretical and measured surface roughness values.

**Figure 7:**
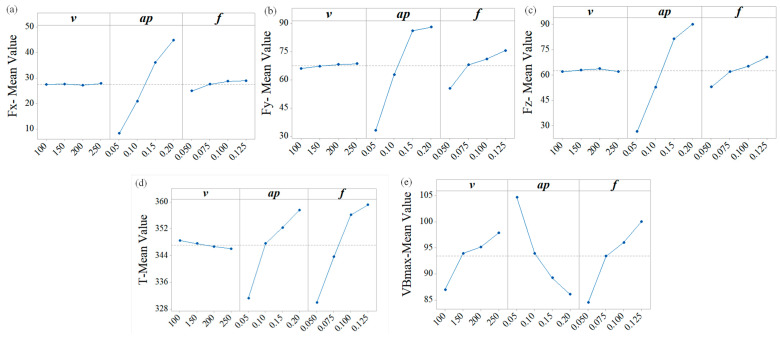
Main effects graphs of the means for orthogonal experimental results (**a**) *F_x_* (**b**) *F_y_* (**c**) *F_z_* (**d**) *T* (**e**) *VB_max_*.

Figure 6 compares the theoretical value Ra* with the measured value *Ra*. All the data indicate that *Ra* is less than 0.8, and most values are around 0.4, which fulfils the precision-machining needs. This proves that hard turning can achieve high-quality surfaces comparable to grinding. When *f* = 0.05 mm·rev^−1^, *Ra* > Ra*. And *Ra* deviates further from Ra* as feed rate increases. This occurs because the high strength of D6AC hardened steel causes significant springback on the machined surface during processing. The tool’s small secondary rake angle compensates for theoretical residual height through the squeezing action of the secondary rake face on the machined surface, thereby improving surface quality. As shown in Figure 7b, higher feed rate *f* values increase radial force *F_y_*, intensifying compression and flattening effects, resulting in *Ra* < Ra*.

**Figure 8:**
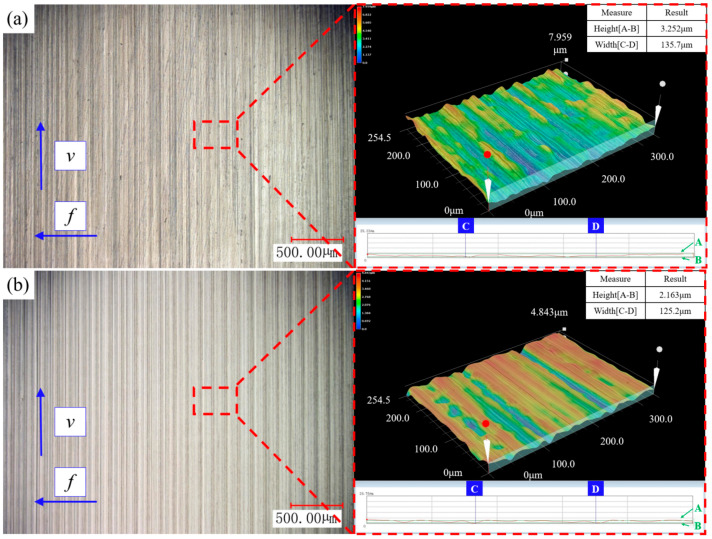
Surface morphology (**a**) *v* = 100 m**·**min^−1^, *a_p_* = 0.05 mm, *f* = 0.05 mm**·**rev^−1^ (**b**) *v* = 200 m**·**min^−1^, *a_p_* = 0.1 mm, *f* = 0.125 mm**·**rev^−1^.

As shown in Figure 8 (3D surface morphology captured using a KEYENCE VHX-1000C digital depth-of-field microscope), comparison of the machined surfaces under the conditions ranked first and last in the grey relational analysis reveals periodic ablation grooves along the cutting speed direction on both surfaces, discoloured by cutting heat. This indicates that, within the experimental range, cutting zone temperatures were sufficiently high to induce thermal oxidation of the surface material. According to condition 1 (*v* = 100 m**·**min^−1^, *a_p_* = 0.05 mm, and *f* = 0.05 mm**·**rev^−1^), the surface quality is relatively poor (*Ra* = 0.65 μm). As shown in Figure 7a, irregularly distributed molten metal deposits visibly adhere to the surface. The small feed rate and depth of cut intensify compressive friction at the cutting edge, which sustains elevated temperatures at the cutting area. Thermal softening of the cutting layer, together with compressive friction, causes plastic bulging and adhesive tearing in the residual zone of the tool tip contour, leading to higher *Ra*. Concurrently, the low thermal capacity of the chips induces significant softening, promoting adhesion to the workpiece surface and forming residues. Under condition 14 (*v* = 200 m**·**min^−1^, *a_p_* = 0.1 mm, and *f* = 0.125 mm**·**rev^−1^), the surface quality is relatively good (*Ra* = 0.42 μm). As shown in Figure 8b, surface grooves are shallow with relatively flat sides. This is attributed to the dominant cutting action of the tool edge under a larger feed rate and depth of cut, with minimal extrusion friction effects. Simultaneously, increased overall heat generation in the cutting area causes thermal weakening of the machined surface on the workpiece. Rebound extrusion of tool surface will produce burnishing effects, and then the measured value *Ra* is less than the theoretical value Ra*.

Within the experimental parameter range of this study, the variation in surface roughness *Ra* is minimal; therefore, its influence is not considered in the subsequent analysis and optimization.

Based on Minitab software (Version 18, Minitab LLC, State College, PA, USA), ANOVA was performed on the orthogonal experimental data. Sequential sums of squares (Seq SS), contribution ratios (CR), *p*-values (P), and the coefficient of determination (R-Sq) are summarized in Table 6. The coefficients of determination (R-Sq) for all response objectives range from 87.51% to 99.82%, indicating that the analysis results possess reliable statistical significance and engineering applicability. The main effects plots of the mean values are presented in Figure 7.

As shown in Table 6 and Figure 7a–c, depth of cut *a_p_* is positively correlated with the cutting forces, whereas *f* has a relatively minor impact, and cutting speed *v* exhibits a negligible effect. The parameter depth of cut *a_p_* governs the width of cut *a_w_*, which determines the volume of material subjected to compression and shear deformation. Because of the high-strength and hardness of the material, with increasing depth of cut *a_p_*, the tool has to overcome more resistance to deformation, which leads to a steep rise in the cutting force. When *a_p_* < 0.15 mm, all three force components increase linearly with depth of cut *a_p_*, because the expanding material volume linearly increases the work required to overcome deformation and friction. When *a_p_* > 0.15 mm, the growth rates of radial force *F_y_* and main cutting force *F_z_* decrease because the rate at which the engaged arc length increases diminishes with further increases in depth of cut *a_p_* and actual cutting-edge angle *κ_re_*. As *f* increases, the undeformed chip thickness *a_c_* increases, leading to a linear rise in the material removal rate. The resulting increases in shear resistance and friction cause radial force *F_y_* and main cutting force *F_z_* to rise with feed rate *f*. Within the examined range, the tool engages the workpiece via an arc-shaped cutting edge, resulting in a relatively small actual cutting-edge angle *κ_re_*. Consequently, axial force *F_x_* is less sensitive to variations in cutting thickness *a_c_*, and increases more gradually with feed rate *f*. The parameter cutting speed *v* exerts the least influence on the three force components. Within *v* = 100–250 m**·**min^−1^, the temperature in the cutting zone remains elevated, causing varying degrees of thermal softening in the workpiece. Consequently, the effect of increasing cutting speed *v* on cutting force can be considered negligible.

The order of influence on cutting temperature *T* is *f* > *a_p_* > *v*. As shown in Figure 7d, cutting temperature *T* decreases slightly with increasing cutting speed *v*. This can be attributed to the increase in shear strain rate in the primary deformation zone as cutting speed *v* rises. Although the high hardness and strength of the material promote heat generation, its low thermal conductivity hinders heat dissipation. Within the investigated range, no obvious adiabatic shear was observed. Reference [27] reported that adiabatic shear in chips occurred at *v* = 60 m·min^−1^ during machining of quenched 34CrNiMo6 high-strength steel (60 HRC, *σ_b_* = 1180 MPa, *λ* = 44.5 W·m^−1^·K^−1^) using a PCBN tool. In the present study, the maximum temperature detected within the thermal imaging field of view decreased slightly at higher cutting speed *v*, likely because the reduced time available for heat conduction altered the measurable temperature distribution. This further confirms the relatively weak influence of cutting speed *v* on cutting temperature *T*. Cutting temperature *T* increases with rising feed rate *f* and depth of cut *a_p_*. For one thing, increasing feed rate *f* and depth of cut *a_p_* enlarges the cutting area and the values of MRR per unit time, consuming more plastic deformation work that is converted into heat energy. For another, the heat capacity at the cutting position increases with rising feed rate *f* and depth of cut *a_p_*. Combined with the low thermal conductivity of D6AC hardened steel, heat becomes concentrated within the first and second deformation zones. The enlarged cutting area further intensifies heat accumulation, resulting in higher cutting temperature *T* values detected in the visible region. Furthermore, feed rate *f* contributes more significantly to cutting temperature *T* than depth of cut *a_p_* because increased feed rate *f* enlarges the cutting area, hence increasing the pressure and frictional coefficient at the tool–chip interface on the rake face, which subsequently raises frictional heat. Meanwhile, increased depth of cut *a_p_* enlarges the chip’s heat dissipation area, allowing for minor heat dispersion despite the material’s poor thermal conductivity.

The order of influence on flank wear width *VB_max_* is: *a_p_* > *f* > *v*. As shown in Figure 7e, flank wear width *VB_max_* decreases with increasing depth of cut *a_p_* but increases with higher cutting speed *v* or feed rate *f*. As shown in Figure 4, wear of the turning tool is predominantly concentrated on the rake surface adjacent to the secondary cutting edge, in the vicinity of the tool nose. During finishing operations, where the relatively small depth of cut *a_p_* results in a small actual cutting-edge angle *κ_re_*, tool wear arises from both the compressive and frictional interactions between the rake face and the workpiece, as well as from the compressive and frictional effects associated with chip flow near the flank region. As the depth of cut *a_p_* increases, the engaged cutting edge length grows. Simultaneously, the width of the squeezing and friction exerted by the workpiece material on the secondary rake face expands with increasing depth of cut *a_p_*. Consequently, the contact stress on the rake face decreases, and flank wear width *VB_max_* diminishes as depth of cut *a_p_* increases. Meanwhile, a larger depth of cut *a_p_* increases the thermal capacity of the cutting zone, mitigating localized high temperatures and enhancing the proportion of heat dissipated by the chip. Abrasive, thermochemical, and adhesive wear are the main mechanisms in hard turning of high-hardness, high-strength materials by PCBN tools [28,29,30]. Increasing depth of cut *a_p_* alleviates flank face wear by reducing localized thermal concentration. Increasing feed rate *f* increases the undeformed chip thickness *a_c_*, simultaneously raising the deformation resistance to be overcome during cutting and the friction between cutting tools, chips, and workpieces. This significantly elevates the contact load on the rake face, accelerating its wear. Simultaneously, an increase in feed rate *f* leads to heat accumulation within the cutting zone. Higher *f* generates frictional heat, which elevates cutting temperature *T* at the contact area of tool–chip on the rake face, further accelerating rake face wear. The effect of cutting speed *v* on flank wear width *VB_max_* is relatively minor, as within the parameter range of this study, its influence on cutting force and temperature is negligible.

### 3.3. Grey Relational Analysis

To optimize the cutting parameters with respect to multiple precision-machining objectives, GRA was employed to balance the conflicting goals of minimizing resultant force *F_∑_*, cutting temperature *T*, surface roughness *Ra*, and flank wear width *VB*_max_ while maximizing MRR. Because all experimental surface roughness *Ra* values were below 0.8 μm and thus satisfied the precision-machining requirement, surface roughness *Ra* was excluded from the final optimization objectives. These parameters were normalized to a scale of 0–1 [31,32], with results shown in Table 7. In precision machining, it is standard practice to prioritize accuracy and stability over efficiency. The “smaller-the-better” targets resultant force *F_∑_*, cutting temperature *T*, and flank wear width *VB*_max_ were each assigned a weight of 0.3, while the “larger-the-better” target MRR received a weight of 0.1. Grey relational coefficients and grades were computed, as shown in Table 7. Parameter combinations with relatively high grey relational grades (GRG, *γi* > 0.65) were found in Experiments 1–4, all corresponding to *f* = 0.05 mm**·**rev^−1^.

### 3.4. Response Surface Analysis and GRG Prediction

**Figure 9:**
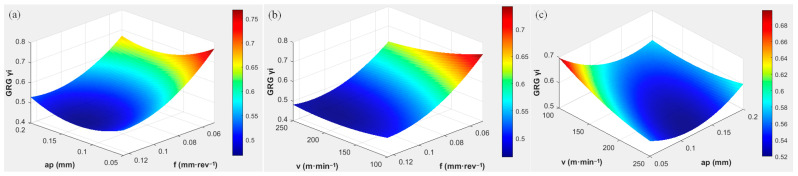
RSM of the GRG (**a**) *v* = 150 m**·**min^−1^ (**b**) *a_p_* = 0.1 mm (**c**) *f* = 0.075 mm·rev^−1^.

RSM was used to establish the relationship between cutting parameters and GRG, as shown in Figure 9. A corresponding mathematical model was established as shown in Equation (7). The coefficients obtained from multiple linear regression are listed in Table 8. The conclusions drawn from the model are consistent with the GRA results, and its high R^2^ value of approximately 0.96 indicates high reliability of the predicted values.

(7) $$\gamma_{i}=K_{1}+K_{2}v+K_{3}a_{p}+K_{4}f+K_{5}va_{p}+K_{6}vf+K_{7}a_{p}f+K_{8}v^{2}+K_{9}{a_{p}}^{2}+K_{10}f^{2}$$

The results demonstrate that the ranking of the effects of cutting parameters on *γ_i_* is *f* (CR = 79.67%) > *a_p_* (CR = 20.31%) > *v* (CR ≈ 0%). This significant discrepancy arises from the thermomechanical coupling conditions present during the cutting process. For precision hard turning of D6AC quenched steel using PCBN tools, the core strategy is strict low *f* control to minimize thermal load.

## 4. Limitations of the Study

This study has several limitations. First, all experiments were conducted under dry cutting conditions; the effects of minimum quantity lubrication (MQL) or cryogenic cooling were not investigated, which may further improve tool life and surface integrity. Second, only one grade and geometry of PCBN insert (ISCAR WNGA 080404T-MC) was tested, and the results may vary with different tool materials or edge preparations. Third, the predictive models were developed within a specific range of cutting parameters (*v* = 100–250 m·min^−1^, *a_p_* = 0.05–0.2 mm, *f* = 0.05–0.125 mm·rev^−1^) and for a single workpiece hardness (47–49 HRC); extrapolation beyond these conditions requires further validation.

## 5. Conclusions

This study employs PCBN tools for precision turning of D6AC hardened steel. To enhance quality while reducing efficiency and costs, Taguchi orthogonal experiments were conducted to analyze the cutting mechanism, followed by multi-objective optimization. Within *v* = 100–250 mm**·**rev^−1^, *a_p_* = 0.05–0.2 mm, and *f* = 0.05–0.125 mm**·**rev^−1^, the conclusions are summarized as follows:

(1) Predictive models for axial force *F_x_*, radial force *F_y_*, main cutting force *F_z_*, *T*, and flank wear width *VB_max_* were established with high accuracy, yielding R^2^ values ranging from 0.86 to 0.97. Notably, the R^2^ values for cutting forces and flank wear width *VB_max_* exceeded 0.9, indicating exceptional performance.
(2) Performance metrics are least affected by cutting speed *v*, as thermal softening occurs throughout the cutting process within the study range. The influence sequence of cutting forces follows *a_p_* > *f* > *v*. Prioritizing a smaller depth of cut *a_p_* followed by a smaller *f* reduces cutting forces. The influence sequence for cutting temperature *T* is *f* > *a_p_* > *v*; selecting a smaller *f* followed by a smaller depth of cut *a_p_* suppresses cutting temperature *T*. Flank wear width *VB_max_* follows *a_p_* > *f* > *v*; prioritizing a larger depth of cut *a_p_* followed by a smaller *f* slows tool wear.
(3) All measured surface roughness *Ra* was below 0.8 μm, with most surface roughness *Ra* ≈ 0.4 μm, confirming that hard turning can achieve excellent surface finish. If the feed rate *f* is greater than or equal to 0.075 mm**·**rev^−1^, the measured value *Ra* is lower than the theoretical value Ra*, and the difference increases with rising *f*. This results from the “extrusion–flattening” effect imposed by the tool on the machined surface.
(4) The rank of GRG and model of RSM both identify optimal parameters as *v* = 100 m**·**min^−1^, *a_p_* = 0.05 mm, *f* = 0.05 mm**·**rev^−1^, and high-GRG combinations are found at *f* = 0.05 mm**·**rev^−1^. Parameter selection prioritizes small feed rate *f*, then appropriate depth of cut *a_p_*, with cutting speed *v* determined via GRG model based on machine/tool performance.

Future work should extend the present framework to wider cutting domains, different PCBN grades and edge preparations, longer-duration tool-life experiments, and dry, MQL, or coolant-assisted cutting conditions, so as to build a more general optimization database for the precision machining of D6AC steel and related high-strength materials.

## Figures and Tables

**Table 1:** Chemical composition of D6AC hardened steel (wt.%).

Element	C	Mn	Si	V	S	P	Ni	Cr	Mo
Content	0.48	0.83	0.3	0.1	0.00084	0.006	0.6	1.07	1.04

**Table 2:** Material properties of D6AC hardened steel.

Physical Property	Value	Physical Property	Value
Yield strength, *σ_s_/*(MPa)	1450	Tensile strength, *σ_b_/*(MPa)	2030
Hardness	49HRC	Thermal conductivity, *λ*/(W·m^−1^·K^−1^)	24
Elongation after fracture, *δ*/(%)	11	Elastic modulus, *E*/(GPa)	210

**Table 3:** Geometric parameters of the PCBN tool.

Tool Parameter	Value	Tool Parameter	Value
Working rake angle, *γ*_0_	−6°	Clearance angle, *α*_0_	6°
Principal cutting-edge angle, *κ_r_*	95°	Auxiliary cutting-edge angle, *κ*′*_r_*	5°
Width of negative land, *b_γl_*	0.16 mm	Nose radius, *r_ε_*	0.4 mm

**Table 4:** L16(4^3^) orthogonal array design and experimental results.

No.	*v* (m·min^−1^)	*a_p_* (mm)	*f* (mm·rev^−1^)	*F_x_* (N)	*F_y_* (N)	*F_z_* (N)	*F_∑_* (N)	*T* (°C)	*VB*_max_ (μm)	*Ra* (μm)	MRR (mm^3^·min^−1^)
1	100	0.05	0.05	6.0	19.5	16.0	25.9	315.2	87.45	0.65	250
2	150	0.1	0.05	18.3	51.8	44.6	70.8	339.8	83.06	0.40	750
3	200	0.15	0.05	32.5	73.6	73.4	108.9	331.6	83.28	0.38	1500
4	250	0.2	0.05	42.3	76.1	77.7	116.7	332.8	82.64	0.65	2500
5	150	0.05	0.075	8.3	32.7	26.5	42.9	327.7	108.52	0.43	562.5
6	100	0.1	0.075	20.2	60.3	49.4	80.5	336.4	86.47	0.24	750
7	250	0.15	0.075	36.9	88.2	80.0	124.7	350.5	92.00	0.33	2812.5
8	200	0.2	0.075	44.3	90.2	91.8	136.1	359.6	86.49	0.40	3000
9	200	0.05	0.1	8.7	36.8	29.1	47.7	333.3	107.67	0.45	1000
10	250	0.1	0.1	21.9	66.5	56.2	89.8	352.0	101.72	0.40	2500
11	100	0.15	0.1	37.4	89.4	83.7	128.1	371.7	86.69	0.40	1500
12	150	0.2	0.1	46.4	91.2	92.1	137.7	367.3	87.94	0.62	3000
13	250	0.05	0.125	9.7	42.9	34.2	55.7	348.5	115.12	0.37	1562.5
14	200	0.1	0.125	22.8	71.6	60.7	96.6	362.0	103.05	0.42	2500
15	150	0.15	0.125	37.1	93.0	88.5	133.6	355.4	94.76	0.51	2812.5
16	100	0.2	0.125	45.8	94.4	98.7	144.1	370.4	87.13	0.58	2500

**Table 5:** Evaluation metrics for the regression prediction models.

Parameter	$F_{x}$	$F_{y}$	$F_{z}$	*T*	*VB*_max_
R^2^	0.97	0.90	0.95	0.86	0.95
MRE	5.90%	9.01%	7.85%	1.46%	1.22%

**Table 6:** ANOVA for mean values.

Target	Parameter	*v*	*a_p_*	*f*	Target	Parameter	*v*	*a_p_*	*f*
*F_x_*	Seq SS	0.85	3143.92	41.73	*T*	Seq SS	14.00	1558.09	2136.44
	CR	0.03%	98.58%	1.31%		CR	0.33%	36.77%	50.42%
	P	0.637	≪0.001	0.001		P	0.982	0.032	0.016
	R-Sq	99.91%				R-Sq	87.51%		
*F_y_*	Seq SS	15.09	7919.02	903.24	*VB*_max_	Seq SS	259.08	797.21	547.57
	CR	0.17%	89.49%	10.21%		CR	16.12%	49.47%	32.34%
	P	0.143	≪0.001	≪0.001		P	0.004	≪0.001	0.001
	R-Sq	99.87%				R-Sq	97.76%		
*F_z_*	Seq SS	8.7	10,050.6	656.0					
	CR	0.08%	93.62%	6.11%					
	P	0.519	≪0.001	≪0.001					
	R-Sq	99.81%							

**Table 7:** GRA data.

No.	Normalized Values				GRC				GRG γi	Rank
	*F_∑_*_norm	*T*_norm	*VB*_max__norm	MRR_norm	*ζ*(*F_∑_*)	*ζ*(*T*)	*ζ*(*VB*_max_)	*ζ*(MRR)		
1	1	1	0.85	0	1	1	0.77	0.33	0.865	1
2	0.62	0.56	0.99	0.18	0.57	0.53	0.94	0.38	0.661	3
3	0.30	0.71	0.98	0.45	0.52	0.63	0.96	0.48	0.651	4
4	0.23	0.69	1	0.82	0.39	0.62	1	0.73	0.677	2
5	0.86	0.78	0.20	0.11	0.78	0.69	0.39	0.36	0.593	6
6	0.54	0.62	0.88	0.18	0.52	0.57	0.81	0.38	0.608	5
7	0.16	0.38	0.71	0.93	0.37	0.44	0.63	0.88	0.524	10
8	0.07	0.21	0.88	1	0.35	0.39	0.81	1	0.564	7
9	0.82	0.68	0.23	0.27	0.73	0.61	0.39	0.41	0.561	8
10	0.46	0.35	0.41	0.82	0.48	0.43	0.46	0.73	0.486	15
11	0.14	0	0.88	0.45	0.37	0.33	0.80	0.48	0.498	12
12	0.05	0.08	0.84	1	0.35	0.35	0.75	1	0.535	9
13	0.75	0.41	0	0.48	0.66	0.46	0.33	0.49	0.486	14
14	0.40	0.17	0.37	0.82	0.46	0.38	0.44	0.73	0.456	16
15	0.09	0.29	0.63	0.93	0.35	0.41	0.57	0.88	0.490	13
16	0	0.02	0.86	0.82	0.33	0.34	0.78	0.73	0.510	11

**Table 8:** Coefficients of the GRG prediction model.

Coefficient	$K_{1}$	$K_{2}$	$K_{3}$	$K_{4}$	$K_{5}$	$K_{6}$	$K_{7}$	$K_{8}$	$K_{9}$	$K_{10}$
Value	1.76	<<0.001	−4.75	−12.54	0.01	0.01	9.03	<<0.001	10.43	42.70

## Data Availability

The original contributions presented in this study are included in the article. Further inquiries can be directed to the corresponding author.
